# Risk stratification in hypertrophic cardiomyopathy

**DOI:** 10.18632/aging.101895

**Published:** 2019-03-28

**Authors:** Milind Desai, Amgad Mentias

**Affiliations:** 1 Heart and Vascular Institute, Department of Cardiovascular Medicine, Cleveland Clinic, Cleveland, OH 44195, USA

**Keywords:** hypertrophic cardio myopathy, fibrosis, outcomes

Hypertrophic cardiomyopathy (HCM) is a common genetic diseases with prevalence of 1 in 200-500 in general population, and it can result in sudden cardiac death (SCD) [1]. Risk stratification in HCM patients is challenging due to heterogeneity in clinical symptoms and phenotypic left ventricle (LV) wall involvement [2]. It was shown recently that the natural history in obstructive HCM is different than non-obstructive HCM [3]. Furthermore, septal reduction therapies such as septal myectomy are known to modify the risk of SCD in these patients [2]. Hence, there is a need to find better tools to quantify risk of SCD in these diverse HCM populations. The current American College of Cardiology/American Heart Association (ACC/AHA) risk model for SCD as well as the European Society of Cardiology (ESC) 5-yeaer SCD risk tool perform better in high risk HCM patients but are questionable in patients in the low/intermediate risk category [1,2,4]. In the study by Maron et al., 60% of HCM patients who had SCD were considered to be low risk patients by the ESC risk score [4]. In another study by Bruder et al., 73% of the SCD events occurred in HCM patients with no conventional risk factors. Furthermore, presence of 2 risk factors failed to predict the study endpoint [5]. It was shown that the risk of SCD in HCM patients with no conventional risk factors is 5.9% in 10 years [6].

In our recent study, we sought to address this knowledge gap by evaluating cardiac Magnetic resonance (CMR) in 1423 HCM patients with preserved EF for presence and extent of scar in the LV by late gadolinium enhancement (LGE) [7]. Our study cohort consisted of only low/intermediate risk patients as determined by ACC/AHA and ESC risk scores respectively, as this is the population where risk assessment is not clearly defined yet. We demonstrated that LGE (scar) quantification as % of LV mass significantly reclassified and improved risk prediction of SCD and/or appropriate Intra-cardiac defibrillator (ICD) discharge when added to ESC risk score and ACC/AHA risk model separately. With quadratic spline analysis, we were able to suggest a cutoff value of 15% LGE of LV mass where the risk of SCD increases exponentially and significantly. This cutoff value was similar when obstructive and non-obstructive HCM patients were analyzed separately in our study. In fact, we showed that patients who are considered low risk by conventional risk models who have LGE% less than 15% have very low rate of adverse events in follow up. Furthermore, Septal myectomy in the obstructed HCM subgroup was independently and significantly associated with improved survival. In this subgroup of patients, the cutoff of LGE% associated with increased risk of SCD postoperatively was 25%, which suggests that septal myectomy modulate the risk profile in these patients. We propose that LGE quantification offers a potential tool for better risk stratification in low risk HCM patients.

In the current AHA/ACC guidelines, CMR carries a weak recommendation (class IIB) (Level of evidence C) for use to assess presence of LGE in case of patients with inconclusive SCD risk stratification by conventional factors [2]. Several prior reports have investigated the role of LGE in predicting adverse events in HCM patients. In the study by Chen et al., extent of LGE was a significant predictor of SCD and appropriate ICD discharges with HR 1.46/10% increase in LGE [8]. This study also suggested a cutoff value of 15% of LV mass where risk of SCD is increased by at least two fold. The strengths of our study include that we validated the role of LGE quantification in risk stratification in both types of HCM, obstructive and non-obstructive. Furthermore, we validated that the same tool could potentially be used in obstructive patients who undergo septal myectomy.
